# Lower Extremity Function following Partial Calcanectomy in High-Risk Limb Salvage Patients

**DOI:** 10.1155/2015/432164

**Published:** 2015-01-27

**Authors:** Noah G. Oliver, John S. Steinberg, Kelly Powers, Karen K. Evans, Paul J. Kim, Christopher E. Attinger

**Affiliations:** ^1^Department of Plastic Surgery, Medstar Georgetown University Hospital, 3800 Reservoir Road Northwest, Washington, DC 20007, USA; ^2^Department of Plastic Surgery, Georgetown University School of Medicine, 3800 Reservoir Road Northwest, Washington, DC 20007, USA; ^3^Associated Podiatrists of Greenwich, 46 Milbank Avenue, Greenwich, CT 06830, USA

## Abstract

Partial calcanectomy (PC) is an established limb salvage procedure for treatment of deep heel ulceration with concomitant calcaneal osteomyelitis. The purpose of this study is to determine if a relationship exists between the amount of calcaneus removed during PC and the resulting lower extremity function and limb salvage outcomes. Consecutive PC patients were retrospectively divided into two cohorts defined by the amount of calcaneus resected before wound closure: patients in cohort 1 retained = 50% of calcaneus, while patients in cohort 2 underwent resection of >50% of the calcaneus. The Lower Extremity Function Scale (LEFS) was used to assess postoperative lower extremity function. The average amount of calcaneus resected was 13% ± 9.2 (1–39%) and 74% ± 19.5 (51–100) in cohorts 1 and 2, respectively (*P* < 0.0001). Below knee amputation was performed in 7 (28%) and 5 (29%) of subjects in cohorts 1 and 2, respectively (*P* = 1.0). The average LEFS score was 33.9 ± 15.0 for subjects in cohort 1 and 36.2 ± 19.9 for the subjects cohort 2 (*P* = 0.8257) which correlates to “moderate to quite a bit of difficulty.” Our study suggests that regardless of the amount of calcaneus resected, PC provides a viable treatment option for high-risk patients with calcaneal osteomyelitis.

## 1. Introduction

Partial calcanectomy (PC) is a well-established treatment for heel ulcers with concomitant calcaneal osteomyelitis (OM) [1–3]. Heel ulcers usually result from a combination of chronic pressure, neuropathy, and peripheral arterial disease in patients with multiple high-risk comorbidities including diabetes [1–3]. In cases of longstanding ulceration or gangrene calcaneal, OM results from continuous spread of infection [4]. The heel is the second most common location for a pressure ulcer and the most likely location for an ulcer to result in lower extremity (LE) amputation [5, 6].

Foot ulcers precede more than 80% of lower extremity amputations, and diabetic foot infections are the leading cause of nontraumatic LE amputations [7]. A recent study found that major LE amputations were performed in 52% of patients with OM of the heel [4]. Treatment goals in these challenging and limb threatening cases include eradication of infection, durable soft-tissue coverage, and maximizing lower extremity function [8, 9]. Since its original description by Gaenslen in 1931 [10], the PC has demonstrated the ability to accomplish these goals and provide a viable treatment alternative to amputation for patients with heel ulcers and calcaneal OM [1–3, 11–18].

Limb salvage goals at our institution are increasingly focused on maximizing patient function and quality of life [8]. While a successful PC can lead to limb preservation, few studies have addressed the impact of PC on lower extremity function and none of these studies have assessed function using a validated scoring system [2].

Although PC is intended to allow unassisted ambulation and has proven a valuable limb salvage option for our patients, we recognize that inherent functional consequences exist, including weakness and gait dysfunction [9, 11]. When attempting to predict patient outcomes for a PC candidate, we have long assumed that some of the structural and functional complications might be influenced by the amount of calcaneus resected. We are unaware of any previous study evaluating the amount of calcaneus removed as a potential variable contributing to postoperative function and outcomes.

We hypothesize that patients with a PC requiring resection of less than half of the calcaneus should function better than patients requiring resection of greater than half of the calcaneus. The purpose of this retrospective study is to evaluate lower extremity function with a validated scoring system and to determine if functional and limb salvage outcomes are influenced by the amount of calcaneus resected in patients with a partial calcanectomy.

## 2. Methods

**Figure 1:**
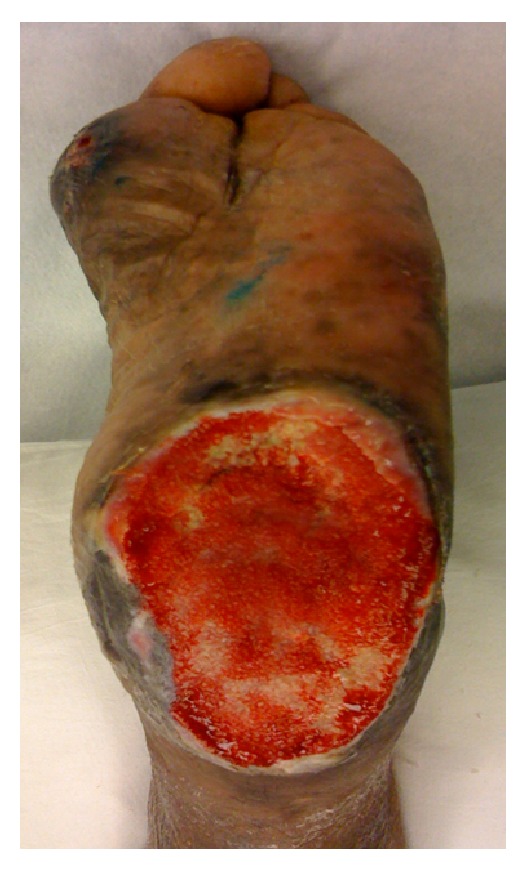
Heel ulcer with osteomyelitis.

A retrospective review was performed of all patients who underwent a partial calcanectomy at a single institution (Georgetown University Hospital) and were treated by four surgeons (Christopher E. Attinger, John S. Steinberg, Karen K. Evans, and Paul J. Kim) between the years 2005 and 2010. The following are the inclusion criteria for this study: PC performed for calcaneal osteomyelitis with heel wound (Figure 1), preoperative and postoperative lateral foot radiograph, and a minimum of 12-month follow-up. Subjects were excluded from the study if they required proximal reamputation on the same hospital admission and before closure of the PC.

**Figure 2:**
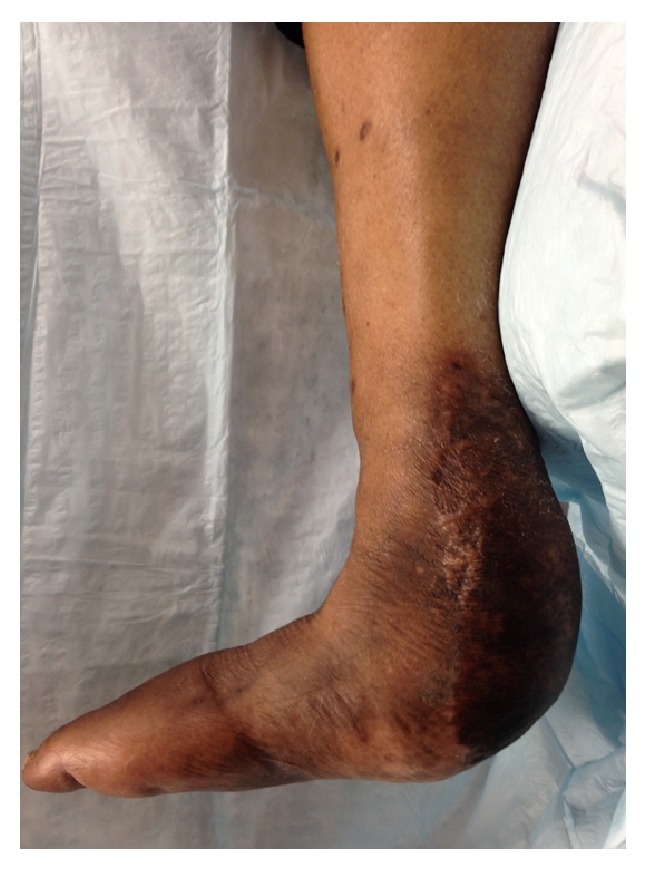
Category 2 partial calcanectomy.

All PCs were staged to allow confirmation of infection eradication before wound closure. A Gaenslen type incision was used to excise the heel ulcer, create durable full thickness flaps, and expose the pathologic calcaneus. All of the infected and nonviable bone and soft tissue were resected with a wide margin to the level of healthy bleeding calcaneus. Once postdebridement wound cultures and bone biopsy of the clean margin were negative for infection and clinical signs of infection had resolved, the patient was brought back to the operating room for delayed primary closure (Figure 2). The calcaneal resection was performed at an oblique angle, parallel to the posterior facet of the subtalar joint, and modified as needed to allow tension-free wound closure. The postoperative protocol was individualized to patient requirements and generally included slow progression to full weight bearing, beginning at 6 to 10 weeks after surgery, in a custom molded orthotic.

**Figure 3:**
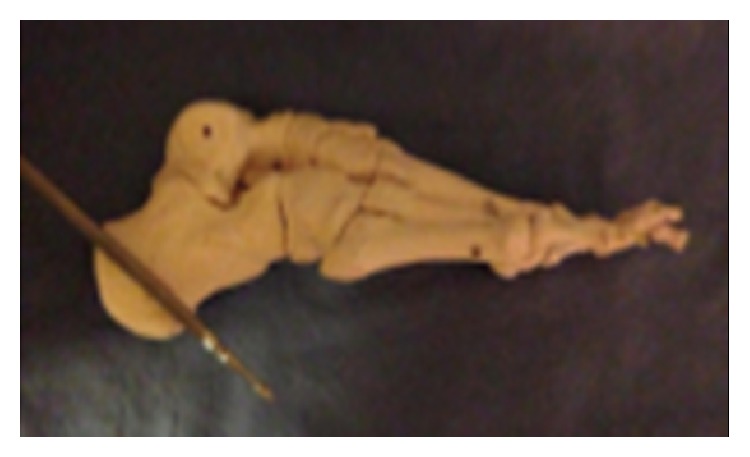
Category 1 partial calcanectomy: ≤50% resected.

**Figure 4:**
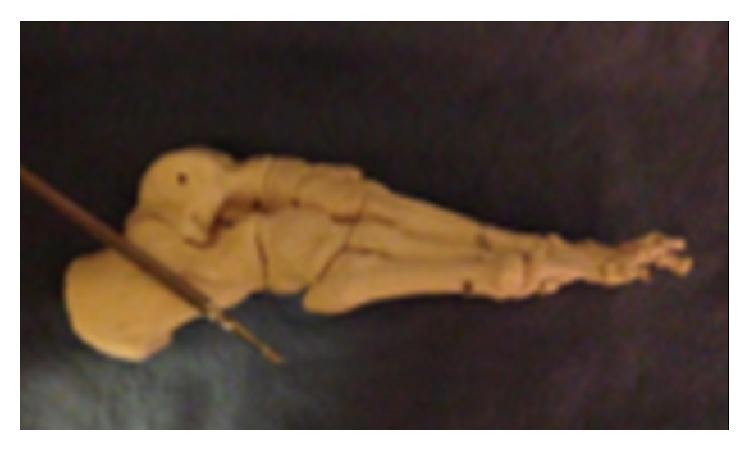
Category 2 partial calcanectomy: >50% resected.

**Figure 5:**
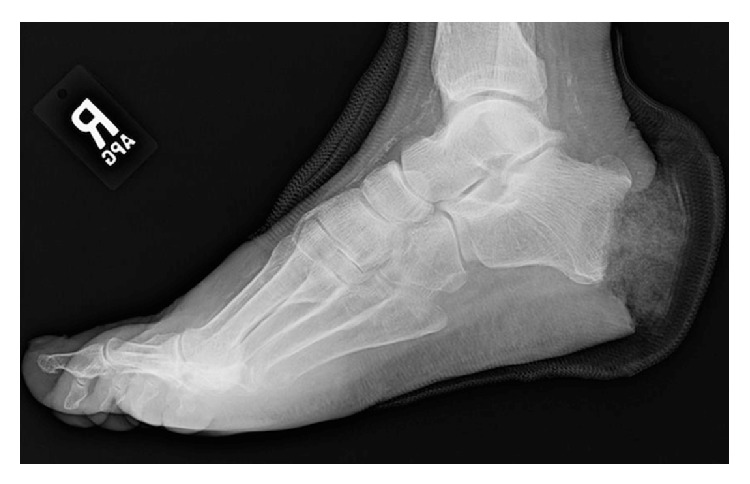
Category 1 partial calcanectomy.

**Figure 6:**
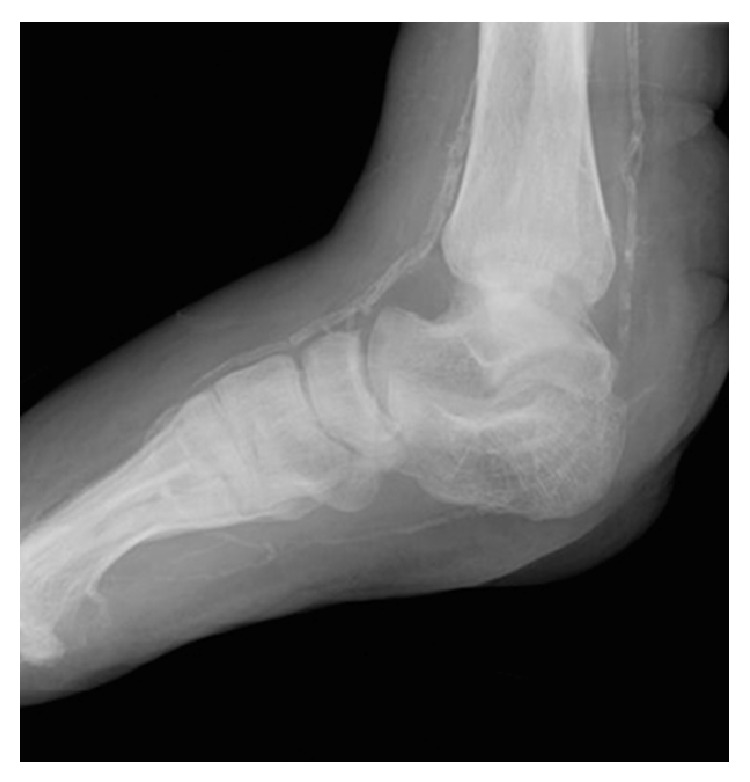
Category 2 partial calcanectomy.

The amount of calcaneus resected during PC was calculated from the difference in two-dimensional surface area, comparing preoperative and postoperative lateral radiographs using PictZar digital planimetry software (Elmwood Park, NJ). The subjects were divided into two cohorts corresponding with the amount of calcaneus resected before wound closure. Patients in cohort 1 (referred to as category 1 PC) retained ≤50% of calcaneus, while patients in cohort 2 (referred to as category 2 PC) underwent resection of >50% of the calcaneus (Figures 3–6). Removal of >50% of the calcaneus was used as the level of demarcation because it ensures loss of the entire weight bearing calcaneal tuberosity.

To assess postoperative lower extremity function, patients were asked to complete the Lower Extremity Function Scale (LEFS) during a follow-up clinic visit or via phone interview [19, 20]. The LEFS is a self-report measure designed to assess the functional status of patients with any musculoskeletal condition related to the lower extremity by asking the patient to rate their perceived difficulties in activities of daily living [19, 20]. Total scores range from 0 to 80, with a higher score indicating better function. The following descriptions are applied to each LEFS score: 0–19:* “Extreme Difficulty or Unable to Perform”*; 20–39:* “Quite A Bit Of Difficulty”*; 40–59:* “Moderate Difficulty”*; 60–79:* “A Little Bit of Difficulty”*; 80:* “No Difficulty”* (perfect score).

Patient demographics, past medical history, and need for PC revision or below-knee amputation were recorded from the medical record. Patency of the medial and lateral calcaneal arteries was determined by evaluating available angiograms performed within 12 months of the PC. To determine if a relationship exists among the amounts of calcaneus resected, lower extremity function, and outcomes following partial calcanectomy, we compared the LEFS scores and below-knee amputation (BKA) rates between cohorts of patients that underwent a category 1 or category 2 type partial calcanectomy.

Statistical analysis included baseline characteristics compared utilizing two-sample *t*-test for continuous variables and Fisher exact tests for categorical variables. A *P* value of < 0.05 was considered statistically significant. The frequencies and percentages for categorical variables and the means and standard deviations for the continuous variables are provided in the following tables. The Georgetown University Medical Center Institutional Review Board approved this study.

## 3. Results

Forty-two patients met inclusion criteria. Forty-five patients were excluded most frequently due to lack of radiographs or follow-up. Cohort 1 included 25/42 subjects with less than 50% of calcaneus resected and cohort 2 included 17/42 subjects with 50% or more of calcaneus resected. Average follow-up was 41 ± 26 months.

In cohort 1, the average subject age was 61 ± 16.6 years, 17/25 (68%) were male, and 8/25 (32%) were female; average body mass index (BMI) was 30 ± 8.6. Comorbidities and medical history included 20 subjects with diabetes mellitus, 13 subjects with peripheral vascular disease (PVD), and 9 subjects with end stage renal disease (ESRD). In cohort 2, the average subject age was 64 ± 16.0 years, 13 (76%) were male, and 4 (24%) were female; average body mass index (BMI) was 31 ± 6.5. Comorbidities and medical history included 15 subjects with diabetes mellitus, 6 subjects with peripheral vascular disease (PVD), and 14 subjects with end stage renal disease (ESRD). No significant difference was identified comparing demographics or medical history in subjects in cohorts 1 and 2 (*P* > 0.05) (Table 1).

Angiograms performed on 10 patients in cohorts 1 and 2 revealed minimal difference in the number of subjects in each cohort with intact medial or lateral calcaneal arteries; *P* > 0.1 (Table 2). Vascular intervention was performed in 10/25 (40%) and 8/17 (47%) of subjects in cohorts 1 and 2, respectively (*P* > 0.5). These vascular interventions included endovascular procedures, 24% and 35%, and open bypass, 16% and 12%, for cohorts 1 and 2, respectively (Table 2).

The average amount of calcaneus resected was 13% ± 9.2 (1–39%) and 74% ± 19.5 (51–100) in cohorts 1 and 2, respectively (*P* < 0.0001). After the initial PC, the average number of surgical revisions per subject for dehiscence, infection, or reulceration was 1.8 ± 1.8 and 2 ± 1.3 in cohorts 1 and 2, respectively (*P* = 0.6964). Two subjects in cohort 2 required revision to total calcanectomy. Below-knee amputation was performed in 7 (28%) and 5 (29%) of subjects in cohorts 1 and 2, respectively (*P* = 1.0). The average time to BKA was 11 ± 17 months (range, 2–48 months) and 10 ± 11 months (range, 2–28 months), respectively, in cohorts 1 and 2 (*P* = 0.8319). Postoperative mortality rate was 3 (12%) and 2 (12%) in cohorts 1 and 2, respectively. The LEFS was completed by 12 subjects with a healed PC. The average LEFS score was 33.9 ± 15.0 (range, 19–57) for the 7 subjects evaluated in cohort 1 and 36.2 ± 19.9 (range, 12–65) for the 5 subjects evaluated in cohort 2 (*P* = 0.8257). Outcomes including LEFS score and LE amputation were found to be similar for subjects with both category 1 and category 2 partial calcanectomies (*P* > 0.5) (Table 3).

## 4. Discussion

The PC is a time-honored alternative to BKA for patients with extensive heel wounds with calcaneal osteomyelitis. Limb salvage with PC should aim to provide a foot capable of ambulation when properly accommodated with a shoe filler and/orthotics [2, 9, 11]. When confronted with the difficult decision of attempted limb salvage versus BKA, careful consideration of patient factors and expectations is paramount to select the option that maximizes QOL, function, and independence.

Attinger and Brown [8] propose that postoperative function rather than limb salvage at all cost should be the goal in diabetic limb salvage/amputation. Therefore, the most functional leg for a given patient might be achieved via limb salvage or amputation. For example, “early major amputation may offer the best functional outcome for younger, more active patients, while even a poorly functional salvaged limb can maximize ambulation and quality of life for relatively sedentary patient with diabetes.” An earlier retrospective study at this institution reported higher rates of ambulation (88% versus 64%) and lower mortality rates (20% versus 52%) at two years in a group of patients that underwent successful limb salvage with mid-foot amputation versus BKA [6]. A much larger subsequent study showed that even though BKA amputees with the same comorbidities were able to achieve a 78% ambulatory rate, nonambulators were unable to handle the complexity of using a prosthesis and remained wheelchair bound [21]. The authors emphasize the importance of preserving limb length despite limited function in those patients who would not be able to handle a below-knee amputation.

While the BKA provides active patients with excellent functional results, many patients never learn to ambulate with a prosthesis [22]. Function and ability to perform activities of daily living are severely limited in those patients who are unable to use their prosthesis as they must rely on others as well as a wheelchair existence. Taylor found that vascular amputees were 9.5 times less likely to use their prosthesis if they were nonambulatory preoperatively, 3 times less likely if ambulatory homebound, 2.7 times less likely if older than 60, and 2.4 less likely if suffering from dementia [23]. Patients with diabetes also report that limb loss has a larger negative impact on quality of life than any other complication of diabetes [24].

While return to ambulation after partial calcanectomy is possible provided that the patient has the appropriate orthotics and assisting functional devices, the biomechanical effects of the procedure can be debilitating and may not allow some active patients to achieve their functional goals. Few studies have evaluated lower extremity function following PC. Furthermore, no previous study has evaluated if the amount of calcaneus resected has any effect on postoperative function or limb durability.

The amount of calcaneal resection is primarily dictated by the degree of OM present. Most of the literature describes resection of the calcaneal tuber at an oblique angle approximately 1-2 centimeters posterior to the subtalar joint and calcaneocuboid joint, resulting in removal of at least 50% of the calcaneus [1, 11–14]. This level of PC, with removal of the weight bearing calcaneal tuber and Achilles tendon attachment, ensures disruption to normal LE biomechanics and gait [1]. By doing plantar instead of vertical calcanectomies to attempt to preserve as much of the Achilles tendon attachment as possible, one removes the affected plantar calcaneal cortex only. This carries the risk that the calcaneus fractures because the force of attached Achilles tendon overwhelms the dorsal calcaneal cortex. While a large degree of calcaneal resection is often necessary [11, 14], some patients only require a minimal resection of the posterior or plantar calcaneus [3]. In theory, preserving the majority of the calcaneus, when indicated, could result in more favorable LE biomechanics and functional outcomes as more of the normal weight bearing surface of the foot and Achilles tendon function are maintained. Potential negative effects of minimal resection include failure to resect all of the infected bone and inadequate residual viable soft tissue for closure.

Smith et al. [1] evaluated lower extremity function for 12 patients with large heel ulcers that opted for a PC in lieu of BKA. They found that ambulatory status after PC did not change for the 10 patients with a healed PC. Bollinger and Thordarson [13] performed 22 PCs with removal of the entire posterior tuberosity. Ambulatory status after PC was assessed for available patients using the Volpicelli scale which demonstrated improvement in seven, no change in 10, and decrease in one. Similarly, Baumhauer et al. [12] found that 4/7 patients maintained the same level of ambulation and 3/7 decreased ambulatory ability following total calcanectomy. They suggest that lifelong mobility restrictions are in order to minimize the potential for further complications and submit that unlimited household ambulation is a reasonable functional status following TC.

Baravarian et al. [11] found that posterior compartment muscle strength was decreased by one grade in all patients following PC; however, muscle strength changes were unnoticed by all of the ambulatory patients when placed in proper ankle-foot orthotics. In an earlier review of our PC cases, we noted that ambulation rates were higher in elderly patients with a healed calcanectomy compared to those patients who went on to have a BKA [8].

Schade's systematic review of sixteen retrospective case series included a total of 100 ambulatory patients who underwent PC (*n* = 76) or TC (*n* = 28) for calcaneal OM [2]. According to the review, all patients returned to ambulation and 85% of patients maintained or improved their ambulatory status postoperatively. Her review supports the partial or total calcanectomy as a viable alternative to BKA, demonstrating the ability for most patients to function as an unlimited household ambulator or a limited community ambulator. These studies used the Volpicelli scale to grade functional outcomes [25]. We chose not to use this scale because it is not validated and was developed to evaluate eventual ambulation levels in bilateral LE amputees.

The LEFS is a validated, self-reported measure designed to assess the functional status of patients with any lower extremity musculoskeletal condition by asking the patient to rate their perceived difficulties in activities of daily living [19, 20]. The majority of our patients scored in the “Quite A Bit Of Difficultly” range regardless of the amount of calcaneus removed. We found these outcomes surprising as we expected that patients with a category 1 PC would function better than patients with a category 2 PC. The loss of the Achilles tendon attachment appears to trump the amount of calcaneus resected. These results, while measuring on a different scale, seem to be consistent with previous studies that found PC to result in ambulation at a household or limited community status. Because the LEFS score does not appear to be greatly influenced by the amount of calcaneus resected, the surgeon should not hesitate to perform an aggressive PC when attempting limb salvage.

PC is often performed in comorbid patients with a history of chronic infection, vascular disease, diabetes, and obesity. This scenario contributes to an often lengthy healing course and risk of reulceration, infection, and eventual major amputation. The reported rates of PC failure resulting in BKA vary widely ranging from 0% to 42% [11–18]. Schade's systematic review found that most major postoperative complications occurred in patients with diabetes and were attributed to residual osteomyelitis or soft-tissue infection [2].

Cook et al. [16] retrospectively assessed 42 PCs for factors influencing postoperative healing. They found that the PC wounds were difficult to heal with a total closure rate of 71% at 1 year; average time to full closure was 201 days. Significant preoperative factors associated with delayed healing included infection with methicillin-resistant* Staphylococcus aureus*, vascular disease, albumin level ≤3.0 g/dL, and greater extent of tissue loss. They also found that preoperative PAD was associated with surgical site dehiscence (*P* = 0.001). Lin et al. [26] recently reported their findings on 12 high-risk patients with heel defects and near total artery occlusive disease who were not candidates for vascular reconstruction and therefore underwent PC in lieu of pedicle or free-flap reconstruction. Eight of their 12 PCs healed without further complication. Four (25%) PCs dehisced and were successfully closed with revision of PC or secondary intention.

Our study found a BKA rate of approximately 29% following PC, regardless of the amount of calcaneus resected. We were again surprised by this lack of difference in outcomes given the significant difference in calcaneal resection required between patients in each cohort. While this BKA rate is on the higher side of the published literature, a large percent of our patients suffered from multiple comorbidities (including DM, PVD, and ESRD), making them at high risk for limb loss. Nevertheless, a 30% failure rate is high and every effort should be made to identify factors to prevent such complications.

This study has many shortcomings, including its retrospective nature, small sample size, and limitations in outcomes data. LEFS data was not obtained before the PC procedure and we were unable to measure change in LE function following PC. Unfortunately, we were only able to obtain LEFS scores on 12 subjects with a healed PC. The remaining subjects were lost to follow-up, had undergone BKA, or were deceased at the time of data collection. We were not able to identify in which patients the Achilles tendon was preserved. We assume that the Achilles was nonfunctional in at least all of category 2 subjects. Future studies could compare LEFS scores between subjects with BKA as a result of a failed PC with subjects with stable PC. Prospective, randomized controlled trials comparing diabetic limb salvage with PC and other levels of partial foot amputation to BKA are needed.

## 5. Conclusion

Partial calcanectomy is a viable limb salvage procedure for low activity patients with calcaneal OM and large heel wounds. Lower extremity function and limb salvage appeared to be similar in our patients regardless of the amount of calcaneus resected. Therefore, the surgeon should focus on accomplishing complete eradication of osteomyelitis and a durable soft tissue closure, as the residual amount of calcaneus appears to have minimal effect on function and limb salvage outcomes. Patient goals, QOL, and LE function should be at the center of all limb salvage and amputation discussions. Patients considering PC should understand the risks for delayed healing, limb loss, and limited resulting lower extremity function.

## Figures and Tables

**Table 1:** Demographics and medical history.

	Category 1 PC	Category 2 PC	*P* value
	≤50% calcaneus resected (*n* = 25)	>50% calcaneus resected (*n* = 17)	
Age	61 ± 16.6 (18–89)	64 ± 16.0 (35–93)	0.5630
Male	17 (68%)	13 (76%)	0.7310
Female	8 (32%)	4 (24%)	0.7310
BMI	30 ± 8.6 (17–49)	31 ± 6.5 (22–50)	0.6866
DM	20 (80%)	15 (88%)	1.0000
ESRD	9 (36%)	6 (35%)	1.0000
PVD	13 (52%)	14 (82%)	0.0560

PC: partial calcanectomy; BMI: body mass index; DM: diabetes mellitus; ESRD: end stage renal disease; PVD: peripheral vascular disease.

**Table 2:** Vascular interventions and angiography findings.

	Category 1 PC	Category 2 PC	*P* value
Vascular intervention	10/25 (40%)	8/17 (47%)	0.7549
Endovascular	6/25 (24%)	6/17 (35%)	0.4982
Bypass	4/25 (16%)	2/17 (12%)	1.0000
Medial CA intact	5/10 (50%)	7/10 (70%)	0.6449
Lateral CA intact	6/10 (60%)	2/10 (20%)	0.1698
No intact CA	1/10 (10%)	3/10 (30%)	0.5820

CA: calcaneal artery.

**Table 3:** Outcomes.

	Category 1 PC	Category 2 PC	*P* value
	(*n* = 25)	(*n* = 17)	
% calcaneus resected	13% ± 9.2 (1–39)	74% ± 19.5 (51–100)	<0.0001
Revisions per subject	1.8 ± 1.8 (1–5)	2 ± 1.3 (1–4)	0.6964
BKA	7/25 (28%)	5/17 (29%)	1.0
Months to BKA	11 ± 17 (2–48)	10 ± 11 (2–28)	0.8319
Mortality	3/25 (12%)	2/17 (12%)	1.0
LEFS^*^	33.9 ± 15.0 (19–57)	36.2 ± 19.9 (12–65)	0.8257
Months of follow-up	43 ± 27 (13–98)	38 ± 26 (12–97)	0.5533

BKA: below-knee amputation; LEFS: lower extremity functional scale; ^*^7/25 and 5/17 subjects completed LEFS in cohorts 1 and 2, respectively.
